# Mapping morbidity data and identifying data gaps for EU countries in the Global Burden of Disease 2023 study

**DOI:** 10.1093/pubmed/fdag014

**Published:** 2026-02-26

**Authors:** Rok Hrzic, Grant M A Wyper, Enkeleint A Mechili, Brigid Unim, Alberto Freitas, Milena Santric Milicevic, Ismael Caballero, João Vasco Santos

**Affiliations:** Department of International Health, Care and Public Health Research Institute—CAPHRI, Maastricht University, PO Box 616, 6200 MD, Maastricht, The Netherlands; WHO Collaborating Center for Public Health Leadership and Workforce Development, PO Box 616, 6200 MD, Maastricht, The Netherlands; Clinical and Protecting Health Directorate, Public Health Scotland, 177 Bothwell Street, Glasgow, G2 7ER, UK; School of Health and Wellbeing, University of Glasgow, Clarice Pears Building, 90 Byres Road, Glasgow, G12 8TB, UK; Department of Healthcare, Faculty of Health, University of Vlora, Bulevardi Ismail Qemali street, PO Box 9401, Vlore, Albania; Department of Cardiovascular, Endocrine-metabolic Diseases and Aging, Italian National Institute of Health (ISS), Via Giano della Bella 34, 00162 Rome, Italy; RISE-Health, MEDCIDS, Faculty of Medicine, University of Porto, Alameda Prof. Hernâni Monteiro, 4200-319, Porto, Portugal; Laboratory for Strengthening the Capacity and Performance of the Health System and Workforce for Health Equity, Faculty of Medicine, University of Belgrade, Dr Subotica 15 Street, 11000 Belgrade, Serbia; Information Systems and Technologies Institute, University of Castilla-La Mancha, Moledores s/n, 13071, Ciudad Real, Spain; RISE-Health, MEDCIDS, Faculty of Medicine, University of Porto, Alameda Prof. Hernâni Monteiro, 4200-319, Porto, Portugal; Public Health Unit, ULS Santo António, Rua Saraiva de Carvalho, 4000-520, Porto, Portugal

**Keywords:** morbidity, data availability, European Union, burden of diseases, Global Burden of Diseases

## Abstract

**Background:**

Health data are essential to guide effective public policies, especially in contexts of increasing complexity and the need for rapid responses. The Global Burden of Disease (GBD) study is central to burden of disease estimates, including in the European Union (EU). This study identified gaps in the data underlying morbidity estimates in the 2023 GBD study for 27 EU countries.

**Methods:**

The GBD2023 Sources Tool was used to identify morbidity data sources, which were analysed by country, year, age, and disease. Data coverage was defined as the inclusion of at least one source for all combinations of age, country, year, and disease.

**Results:**

Highest data coverage was observed in Sweden, Austria, and Germany, and lowest in Hungary, Romania, and Bulgaria. There was high coverage for infectious diseases, but considerable gaps for leading morbidity causes, including musculoskeletal (15.9%) and mental disorders (32.9%) and unintentional injuries (28.1%). Data coverage was highest for the 2000s.

**Conclusion:**

The study highlighted variations in EU morbidity data coverage in the GBD2023, suggesting potential differences in estimate reliability. Differences may stem from national health information systems and data-sharing barriers. To enhance estimate accuracy and benchmarking among European health systems, improving data collection and utilization is crucial.

## Background

Health data are essential for public health research and policymaking, particularly as emerging challenges require faster and more precise decision-making regarding improving health system outcomes and informed policy-making regarding the enhancement of service quality and containing costs.^1–3^ One critical use case of health data is modelling the disease burden to better understand the current state of population health and well-being (including progress towards achieving Sustainable Development Goals), enable cross-country comparative health research, assess the effectiveness of public health interventions, and inform future policy and investment priorities.^4^^,^^5^

In the European Union (EU), a wealth of health data is generated by a wide range of entities, from local to international levels. However, the availability and quality of these data vary, especially from a comparative research perspective.^6^ Collaboration between researchers, policymakers, and public health authorities, along with the adoption of standardized data collection protocols, can help mitigate the challenges associated with data availability. The European Health Data Space (EHDS) initiative exemplifies the effort to enhance the interoperability and secure exchange of health data in Europe, thereby fostering an environment conducive to research and evidence-based policymaking. Similarly, the PHIRI project supports data sharing, secondary use of health data, and the development of new methods in data management (e.g. federated research infrastructure).^7^ However, while these efforts will eventually address the underlying problem, data integration from different data holders takes time. In the meantime, other approaches, including statistical modelling, are being used to extract as much value as possible from the existing health data.^8^

The Global Burden of Disease (GBD) collaboration is a prominent example of this approach. The GBD collaboration employs various data processing and modelling techniques to leverage available health data, providing as comprehensive assessment of global health challenges as possible, including metrics for health losses due to mortality, morbidity, and the burden of disease attributable to different pathologies and risk factors.^9^ The GBD study combines Gaussian process regression and Bayesian meta-regression to generate internally consistent estimates of incidence, prevalence, and mortality, borrowing statistical strength across age groups, time periods, and locations.^10^ The estimation process also includes cross-walking to translate disease data collected under different definitions or measurement methods into a common reference definition.^10^ Epidemiological estimates are produced in a hierarchical cascade—first at the global level, then for super-regions, regions, and finally individual countries—so that higher-level estimates constrain and inform those below.^10^ In settings with limited or missing data, country-level estimates therefore reflect the patterns of the parent region and nearby countries where observations exist.

The GBD model results have gained prominence and influence, and are increasingly seen as a key source of information on public health challenges globally, in the European Union, and in individual countries and sub-national regions.^11–14^ However, as with any modelling exercise, insufficient or outdated data can impact the model outputs, introducing uncertainty and potential bias into the estimates. A recent analysis of data on mental health conditions identified significant data gaps in the available health data used by the GBD models, including in high-income settings.^15^ This may imply that the associated GBD estimates should be used with greater caution.

This paper examines the data sources used to generate the morbidity (i.e. nonfatal health outcomes) estimates in the GBD 2023 study for 27 EU countries. We highlight how gaps in data availability differ over time, between EU countries, and by disease category. The purpose of this study is to foster a deeper understanding of the potential bias of GBD 2023 results by examining the extent to which nonfatal health outcome estimates rely on available data versus model assumptions. In addition, it aims to support collaboration between national authorities responsible for health statistics production and researchers by addressing existing data gaps and enhancing policy-making efforts.

## Methods

### Study design

We conducted a first systematic mapping of morbidity data inputs used in the generation of the GBD 2023 study morbidity estimates for countries in the EU, cataloguing each source by country, year, age, and disease category. Using this mapping, we highlighted countries, years, ages, and disease categories with no underlying data sources and discussed their potential impact on the model’s morbidity estimates.

### Data sources

The relevant data sources and their metadata were identified using the Global Burden of Disease Study 2023 Sources Tool, available at https://ghdx.healthdata.org/gbd-2023/sources. The tool enables searching of the metadata describing data sources used in the GBD studies, including the nonfatal health outcomes data sources utilized for the Global Burden of Diseases 2023 study. The extracted data sources encompassed any source of information on disease morbidity, including hospital discharge data, routine disease surveillance, disease registries, society reports, and epidemiological studies examining the incidence or prevalence of specific diseases, for 27 EU countries spanning from 1990 to 2023. For each data source, we extracted the citation, country, disease entity, data provider, period covered, sex, age range covered, and representativeness. The extracted metadata thus provided information on the country, disease entity, and the range of years (1990–2023) and ages (0–99+) covered by each data source.

In GBD studies, disease entities are structured into a hierarchy with four levels: Level 1 includes communicable, maternal, neonatal and nutritional disorders, non-communicable diseases, and injuries; Level 2 defines major cause categories; Level 3 disaggregates the burden into individual disease groups; and Level 4 includes specific diseases or disease subtypes.^10^ This study considered Level 2 and 3 disease categories. Level 1 was deemed too aggregated, and Level 4 too detailed and unevenly available across countries due to varying diagnostic and coding capacities.

### Statistical analysis

We mapped data coverage by country, year, age, and disease entity (at Level 2 or Level 3 of the GBD hierarchy). We defined data coverage as complete (100%) when at least one source provides information for every combination of country, year, disease category, and age (0, 1, …, 99+). Fractional coverage is defined when any age or year (1990–2023) is missing for a combination of country and disease entity.

Let $c\in C$ denote countries, $d\in D$ denote Level 2 and Level 3 disease categories, $y\in Y$ denote years (1990–2023), and $a\in A$ denote single ages (0, 1, 2, …, 99+). We define an indicator variable, $S$, such that:


$$ {S}_{c,d,y,a}=\left\{\!\!\!\begin{array}{c}1\ \mathrm{if}\ \mathrm{at}\ \mathrm{least}\ \mathrm{one}\ \mathrm{source}\ \mathrm{provides}\ \mathrm{data}\ \mathrm{for}\ \left(c,d,y,a\right)\\{}0,\mathrm{otherwise}\end{array}\right. $$

Then, the data coverage for a given country-disease pair is:


$$ {\mathrm{coverage}}_{c,d}=\frac{\sum_{y\ in\ Y}\sum_{a\ in\ A}{S}_{c,d,y,a}}{\left|Y\right|\mid A\mid } $$

This definition enabled us to examine whether each country–year–disease estimate reported in the GBD 2023 study is, in principle, grounded in at least one explicit data source across all ages. This study is a descriptive, exploratory analysis and does not include inferential testing.

**Figure 1:**
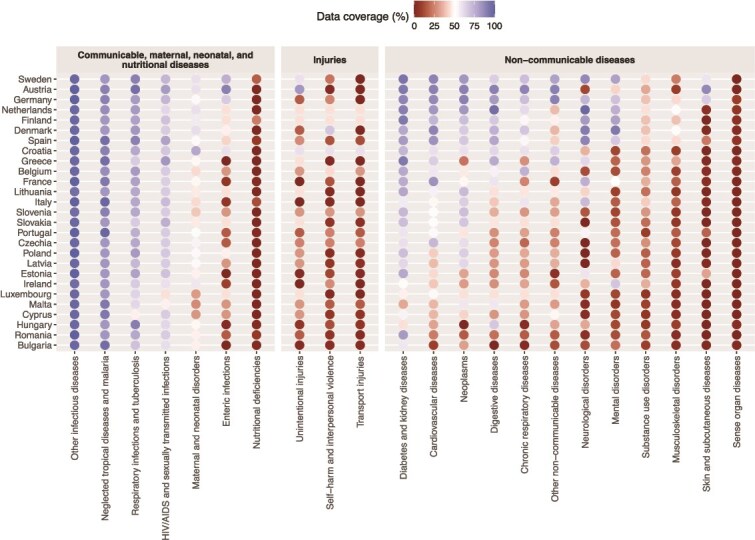
Overview of data coverage by country and disease entity (Level 2; ordered by mean coverage; coverage is defined as at least one available source providing relevant data for 1990–2023 and for ages 0–99+).

Data coverage can be represented as a four-dimensional array indexed by country (*c*), year (*y*), disease entity (*d*), and age (*a*). To make this high-dimensional information interpretable, we summarized the results along different combinations of these dimensions, producing three complementary perspectives on coverage:

Country–disease overview: coverage aggregated across all ages and years, showing the average data coverage for each country and Level 2 disease entity (Fig. 1).

**Figure 2:**
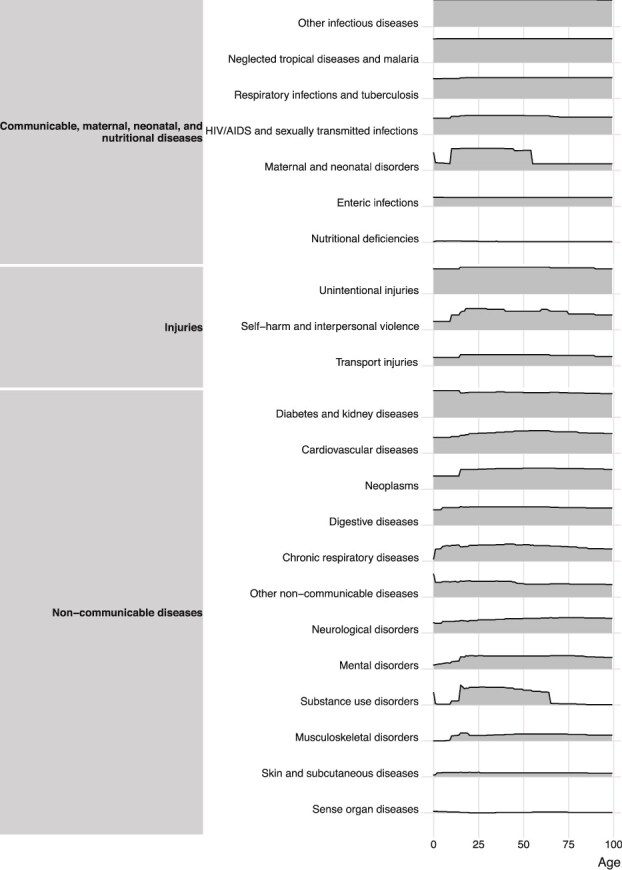
Overview of data coverage by disease entity (Level 2) and age (ordered by mean data coverage across countries, years, and ages), where coverage is defined as at least one available source providing relevant data for each country-year combination from 1990 to 2023.

Disease–age overview: coverage aggregated across all countries and years, showing the average data coverage for each Level 2 disease entity and age (Fig. 2).

Granular disease overview: coverage aggregated across ages, countries, and years, showing the average data coverage for each Level 3 disease entity (Table 1).

**Table 1:** Data coverage by disease entity (Level 3) across all ages (0–99+) and EU countries, 1990–2023.

Cause (Top 30)	Data coverage (%)^a^	Cause (Bottom 30)	Data coverage (%)
Diphtheria	90.1	African trypanosomiasis	0.0
Measles	90.1	Alopecia areata	0.0
Cystic echinococcosis	80.0	Aortic aneurysm	0.0
Schistosomiasis	79.0	Dietary iron deficiency	0.0
Tuberculosis	70.9	Guinea worm disease	0.0
Varicella and herpes zoster	69.1	Hypertensive heart disease	0.0
HIV/AIDS	59.2	Intestinal nematode infections	0.0
Chronic kidney disease	55.6	Lymphatic filariasis	0.0
Ischemic heart disease	42.6	Malaria	0.0
Neonatal disorders	42.4	Onchocerciasis	0.0
Yellow fever	40.8	Other cardiovascular and circulatory diseases	0.0
Sexually transmitted infections excluding HIV	40.0	Other chronic respiratory diseases	0.0
Stroke	33.0	Other digestive diseases	0.0
Non-melanoma skin cancer	32.1	Other intestinal infectious diseases	0.0
Congenital birth defects	30.7	Other mental disorders	0.0
Meningitis	30.5	Other neglected tropical diseases	0.0
Upper digestive system diseases	30.1	Other neurological disorders	0.0
Pancreatitis	28.4	Other nutritional deficiencies	0.0
Diarrheal diseases	28.3	Other sense organ diseases	0.0
Lower respiratory infections	28.3	Other skin and subcutaneous diseases	0.0
Hemoglobinopathies and hemolytic anemias	28.1	Other unspecified infectious diseases	0.0
Interstitial lung disease and pulmonary sarcoidosis	27.3	Pertussis	0.0
Appendicitis	27.1	Protein-energy malnutrition	0.0
Cirrhosis and other chronic liver diseases	27.1	Pruritus	0.0
Diabetes mellitus	26.6	Psoriasis	0.0
Endocrine, metabolic, blood, and immune disorders	26.6	Rabies	0.0
Encephalitis	26.4	Sudden infant death syndrome	0.0
Cardiomyopathy and myocarditis	25.8	Trachoma	0.0
Other neoplasms	25.8	Upper respiratory infections	0.0
Paralytic ileus and intestinal obstruction	25.8	Dengue	0.1

a Coverage is defined as at least one available source providing relevant data.

## Results

Figure 1 and Supplementary Tables S1–S3 illustrate the differences in the data coverage between EU countries by disease entity. The disease categories with the best average data coverage across all countries are infectious diseases, for example, respiratory infections and tuberculosis (75.4%), followed by diabetes and kidney diseases (69.8%), and cardiovascular diseases (56.4%). The poorest coverage was observed for transport injuries (11.0%), nutritional deficiencies (2.6%), and diseases of the sense organs (1.2%).

The countries with the best data coverage across all disease categories and ages in the database were Sweden (60.4%), Austria (59.1%), and Germany (58.6%). On the other end of the scale were Hungary (29.1%), Romania (28.0%), and Bulgaria (24.1%) (see Supplementary Table S2).

Figure 2 highlights differences in data coverage by age and disease category. While the GBD 2023 included enough data sources to provide nearly complete data coverage across all ages for some disease categories, including most infectious diseases categories, unintentional injuries, and diabetes and kidney diseases, there are significant gaps in the coverage of important non-communicable diseases, including chronic respiratory diseases, substance use disorders, and mental disorders in the youngest and oldest age categories.

**Figure 3:**
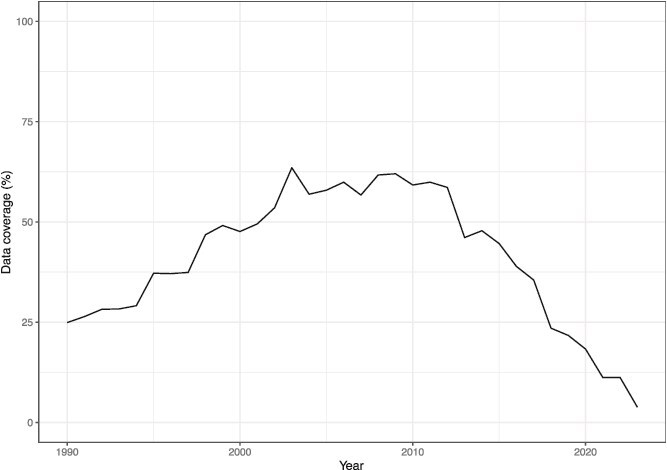
Overview of data coverage by year, where coverage is defined as at least one available source providing relevant data for each country-age-disease entity (Level 2) combination from 1990 to 2023.

Figure 3 and Supplementary Table S3 highlight data coverage over time. Between 1990 and 2003, data coverage increased from 24.9% to 63.5%. However, a continuous decline in data coverage has been observed since 2011, with the early 2020s corresponding to an average data coverage of ~10% across all countries, disease categories, and ages.

Table 1 highlights the top and bottom thirty disease entities (Level 3 in the GBD hierarchy) by data availability for EU countries between 1990 and 2023. Infectious diseases are prevalent in both groups. However, the former group also includes chronic kidney disease, ischaemic heart disease, and stroke. The Supplementary material provides a complete overview by disease.

## Discussion

### Main findings of this study

The primary aim of our study was to examine the extent to which the GBD 2023 study estimates for nonfatal health outcomes rely on available data versus model assumptions. The results highlight disparities in the data coverage underlying the morbidity estimates of the 2023 GBD study among EU countries, across different disease entities, and over time. First, data availability across all disease categories and ages tends to be higher in Western European and Nordic countries compared to other parts of the EU. Second, the data coverage for communicable diseases, diabetes and kidney diseases, and neoplasms is high or moderate across most EU countries, whereas other disease categories exhibit limited or poor coverage. However, we also observed important geographic differences in data coverage within disease categories, particularly in cardiovascular disease. Third and finally, we noted a peak in data coverage for the late 2000s and early 2010s.

### What is already known on this topic and what this study adds

Previous studies have highlighted substantial differences in the availability of morbidity data between EU countries and regions outside the scope of the GBD collaboration.^16^ The geographic disparities in data coverage that we and others observed generally coincide with differences in population health and health system performance previously observed across the EU.^17^ Previous studies have attributed these differences in performance to economic development,^18^^,^^19^ the level and type of healthcare and social welfare spending,^20–22^ and health system governance,^23^ among many other factors. However, we also highlight departures from the expected association between health system performance and the observed data coverage gaps. For example, the data coverage rank for Croatia was higher than expected based on the aforementioned performance measures, whereas the data coverage for France, Italy, and Ireland was lower than expected. Several explanations could be considered, including the idiosyncrasies of data source identification and inclusion in the GBD studies, as well as a partial disconnect between the maturity of a national health research and health information system and other health system performance domains. It is also important to note that the absence of data reuse for the GBD study does not imply that these data are not available at all.

Our findings highlight the low data coverage in the leading three Level 2 causes of morbidity in the EU in 2023 from the GBD 2023 study. An underlying data source was identified for fewer than one-sixth (15.9%) of country-year-age combinations for musculoskeletal disorders and for fewer than one-third for mental disorders (32.9%) and unintentional injuries (28.1%). At the same time, GBD 2023 estimates that these three causes account for 47.5% of all nonfatal health loss across EU member states.^10^ This result highlights the sizable influence of morbidity on EU population health and the necessity to improve certainty around that estimate. Disparities in data coverage between disease entities can be understood in consideration of the differences in legal mandates and data infrastructures for collecting and collating morbidity data. While explicit national and international legal mandates support infectious diseases surveillance, and well-established disease registries exist for cancers, data collection for other disease categories lacks similar impetus and support.^24^

Data coverage improved from under 30% to over 60% between 1990 and the early 2010s. Since GBD 2013, the study has transitioned to a model of producing more regular study iterations, with annual iterations planned starting from GBD 2015.^25^ Our findings highlight a substantial drop-off in data coverage since this strategic move, with data coverage now substantially lower than in 1990, a trend that has been ongoing since 2018. This is a concerning finding, as it highlights that progress in improving data coverage is worsening, yet usage is increasing. This misalignment needs to be addressed by intensifying existing efforts to improve data coverage in the GBD studies. It is unclear to what extent these gaps could be filled in the future. Gaps in the earlier periods of the GBD study (early 1990s) are likely associated with limited data infrastructure and therefore permanent.

### Limitations of this study

This study has several limitations. First, we relied on the GBD 2023 Sources Tool repository to provide a comprehensive overview of the data sources used in creating the estimates reported in the GBD 2023 study. We also assumed that the non-fatal outcomes filter accurately and comprehensively identifies the data sources used for morbidity estimates. Given that this is the official tool produced by the GBD collaboration for this purpose, we do not expect discrepancies. However, we cannot independently verify these assumptions, and it is possible that additional data elements influenced the reported morbidity estimates (e.g. mortality data). Second, the assumption of data coverage used in this study, i.e. one data source per country, year, age, and disease combination, is arbitrary and may be considered too minimal. In an ideal circumstance, one would prefer to have several data sources for each combination to support the estimates. However, since the primary aim of this study was to identify the extent to which certain estimates lack direct empirical support, this minimal definition was deemed appropriate. Third, while we were careful to avoid double-counting data sources across the different levels of the disease hierarchy, we did not independently verify the attribution of individual sources to a particular disease or disease category. This may have resulted in random misattribution and miscounting of data coverage for specific diseases or disease categories. Fourth, the metadata database used in this analysis does not provide information on the type or underlying quality of each data source used in the estimation process. As a result, estimates may be biased due to the limitations inherent in the underlying data despite being supported by one or more data sources. Future research should try to include in-depth investigation of the data sources underlying the GBD estimates given the available metadata. Fifth, the GBD estimation process includes cross-walking to translate disease data collected under different definitions or measurement methods into a common reference definition.^10^ While this may ameliorate the bias derived from using different types of data in the estimation process, it may not do so completely. Future studies should consider investigating the impact of this process on the GBD estimates.

### Recommendations and conclusion

We mapped morbidity data inputs used to generate the GBD 2023 study morbidity estimates for countries in the EU, evaluating data coverage for each combination of country, year, age, and disease category. We found important disparities in data coverage between EU countries, disease categories, and over time. Poorer data coverage tended to cluster in countries in the east and south of the EU, injuries and non-communicable disease categories for which no legal surveillance and reporting mandates and registries exist, and for the years prior to 2005 and after 2012. These data coverage gaps may reflect structural, methodological, and political challenges in national health research and health information infrastructures and data sharing processes between countries and the GBD collaboration. Future research will need to determine the precise nature and causes of these gaps. These data gaps can impact the estimation of the actual burden of disease and, consequently, influence public health policies and the allocation of resources in the EU. The absence of reliable and comprehensive data hampers the ability to assess health inequalities and priorities interventions effectively. A coordinated effort to improve data collection, standardization, and sharing is essential to improving morbidity estimates, which may contribute to reducing health inequalities and supporting more effective public health initiatives. Initiatives like the EHDS offer opportunities to reduce disparities in health data availability between countries and disease categories and contribute significantly to more complete and reliable morbidity data in the EU. Creating comprehensive and comparable estimates of population health and disease burden in the European Union is essential to improve the performance, efficiency, and equity of its health systems.

## Supplementary Material

GBD2023_EUMorbData_supplementary_R1_fda014

## Data Availability

The data underlying this article are available in the Global Burden of Disease Study 2023 Sources Tool, at https://ghdx.healthdata.org/gbd-2023/sources.

## Notes

**Rok Hrzic**, Assistant Professor

**Grant M. A. Wyper**, Principal Epidemiologist

**Enkeleint A. Mechili**, Associate Professor

**Brigid Unim**, Public health Specialist

**Alberto Freitas**, Associate Professor

**Milena Santric Milicevic**, Full Professor in Social Medicine

**Ismael Caballero**, Full Professor in Computer Science

**João Vasco Santos**, Invited Associate Professor
