# Supplementary material for: Mapping morbidity data and identifying data gaps for EU countries in the Global Burden of Disease 2023 study
Source: J Public Health (Oxf). 2026 Feb 26;48(2):391–8. doi: 10.1093/pubmed/fdag014 (PMC13268799; doi:10.1093/pubmed/fdag014)
Supplement: GBD2023_EUMorbData_supplementary_R1_fda014 [file gbd2023_eumorbdata_supplementary_r1_fda014.docx]

**Supplementary material**

**Mapping morbidity data and identifying data gaps for EU countries in the Global Burden of Disease 2023 study**

Rok Hrzic*, Grant M A Wyper, Enkeleint A. Mechili, Brigid Unim, Alberto Freitas, Milena Santric Milicevic, Ismael Caballero, and João Vasco Santos

*Corresponding author’s contact information:

[r.hrzic@maastrichtuniversity.nl](mailto:r.hrzic@maastrichtuniversity.nl)

**Supplementary Table 1 Data coverage by cause category (Level 2) across all ages (0-99+) and EU countries, 1990-2023**

| **Cause category** | **Data coverage (%)^a^** |
| --- | --- |
| Other infectious diseases | 97.7 |
| Neglected tropical diseases and malaria | 86.6 |
| Respiratory infections and tuberculosis | 75.4 |
| Diabetes and kidney diseases | 69.8 |
| HIV/AIDS and sexually transmitted infections | 65.9 |
| Cardiovascular diseases | 56.4 |
| Neoplasms | 54.3 |
| Digestive diseases | 49.5 |
| Maternal and neonatal disorders | 49.1 |
| Chronic respiratory diseases | 41.2 |
| Other non-communicable diseases | 39.8 |
| Neurological disorders | 38.6 |
| Mental disorders | 32.9 |
| Enteric infections | 31.7 |
| Unintentional injuries | 28.1 |
| Substance use disorders | 24.6 |
| Self-harm and interpersonal violence | 18.6 |
| Musculoskeletal disorders | 15.9 |
| Skin and subcutaneous diseases | 11.0 |
| Transport injuries | 11.0 |
| Nutritional deficiencies | 2.6 |
| Sense organ diseases | 1.2 |
| ^a^Coverage is defined as at least one available source providing relevant data; | |

**Supplementary Table 2 Data coverage by EU country across all ages (0-99+) and cause categories (Level 2), 1990-2023**

| **Country** | **Data coverage (%)^a^** | **Country** | **Data coverage (%)^a^** |
| --- | --- | --- | --- |
| Sweden | 60.4 | Slovakia | 37.2 |
| Austria | 59.1 | Portugal | 36.7 |
| Germany | 58.6 | Czechia | 35.3 |
| Netherlands | 58.2 | Poland | 35.1 |
| Finland | 55.4 | Latvia | 34.2 |
| Denmark | 54.1 | Estonia | 33.2 |
| Spain | 53.6 | Ireland | 33.2 |
| Croatia | 49.5 | Luxembourg | 32.1 |
| Greece | 42.1 | Malta | 30.7 |
| Belgium | 41.5 | Cyprus | 30.0 |
| France | 39.9 | Hungary | 29.1 |
| Lithuania | 39.4 | Romania | 28.0 |
| Italy | 38.5 | Bulgaria | 24.1 |
| Slovenia | 37.4 |  |  |
| ^a^Coverage is defined as at least one available source providing relevant data; | | | |

**Supplementary Table 3 Data coverage by year across all ages (0-99+), cause categories, and EU countries**

| **Year** | **Data coverage (%)^a^** | **Year** | **Data coverage (%)^a^** |
| --- | --- | --- | --- |
| 1990 | 24.9 | 2007 | 56.7 |
| 1991 | 26.4 | 2008 | 61.7 |
| 1992 | 28.2 | 2009 | 62.0 |
| 1993 | 28.3 | 2010 | 59.2 |
| 1994 | 29.1 | 2011 | 59.9 |
| 1995 | 37.2 | 2012 | 58.6 |
| 1996 | 37.1 | 2013 | 46.1 |
| 1997 | 37.4 | 2014 | 47.8 |
| 1998 | 46.8 | 2015 | 44.6 |
| 1999 | 49.1 | 2016 | 38.9 |
| 2000 | 47.6 | 2017 | 35.5 |
| 2001 | 49.5 | 2018 | 23.5 |
| 2002 | 53.5 | 2019 | 21.7 |
| 2003 | 63.5 | 2020 | 18.3 |
| 2004 | 56.9 | 2021 | 11.2 |
| 2005 | 57.9 | 2022 | 11.2 |
| 2006 | 59.9 | 2023 | 3.8 |
| ^a^Coverage is defined as at least one available source providing relevant data; | | | |

**Supplementary Table 4 Supplementary Table 4 Data coverage across all Level 3 GBD causes, ages (0-99+), and EU countries, 1990-2023**

| **Cause** | **Data coverage (%)^a^** |
| --- | --- |
| Diphtheria | 90.1 |
| Measles | 90.1 |
| Cystic echinococcosis | 80.0 |
| Schistosomiasis | 79.0 |
| Tuberculosis | 70.9 |
| Varicella and herpes zoster | 69.1 |
| HIV/AIDS | 59.2 |
| Chronic kidney disease | 55.6 |
| Ischemic heart disease | 42.6 |
| Neonatal disorders | 42.4 |
| Yellow fever | 40.8 |
| Sexually transmitted infections excluding HIV | 40.0 |
| Stroke | 33.0 |
| Non-melanoma skin cancer | 32.1 |
| Congenital birth defects | 30.7 |
| Meningitis | 30.5 |
| Upper digestive system diseases | 30.1 |
| Pancreatitis | 28.4 |
| Diarrheal diseases | 28.3 |
| Lower respiratory infections | 28.3 |
| Hemoglobinopathies and hemolytic anemias | 28.1 |
| Interstitial lung disease and pulmonary sarcoidosis | 27.3 |
| Appendicitis | 27.1 |
| Cirrhosis and other chronic liver diseases | 27.1 |
| Diabetes mellitus | 26.6 |
| Endocrine, metabolic, blood, and immune disorders | 26.6 |
| Encephalitis | 26.4 |
| Cardiomyopathy and myocarditis | 25.8 |
| Other neoplasms | 25.8 |
| Paralytic ileus and intestinal obstruction | 25.8 |
| Urinary diseases and male infertility | 25.7 |
| Gallbladder and biliary diseases | 25.6 |
| Pneumoconiosis | 25.6 |
| Acute glomerulonephritis | 25.3 |
| Adverse effects of medical treatment | 25.3 |
| Inguinal, femoral, and abdominal hernia | 25.3 |
| Vascular intestinal disorders | 25.3 |
| Rheumatic heart disease | 24.7 |
| Inflammatory bowel disease | 23.7 |
| Gynecological diseases | 23.5 |
| Multiple sclerosis | 23.4 |
| Drug use disorders | 23.3 |
| Leishmaniasis | 23.3 |
| Endocarditis | 22.7 |
| Maternal disorders | 20.3 |
| Depressive disorders | 20.2 |
| Acute hepatitis | 19.4 |
| Atrial fibrillation and flutter | 19.0 |
| Alzheimer's disease and other dementias | 16.7 |
| Asthma | 16.5 |
| Parkinson's disease | 15.6 |
| Self-harm | 15.4 |
| Non-rheumatic valvular heart disease | 15.1 |
| Breast cancer | 13.6 |
| Liver cancer | 12.4 |
| Colon and rectum cancer | 12.3 |
| Brain and central nervous system cancer | 12.1 |
| Chagas disease | 12.0 |
| Non-Hodgkin lymphoma | 11.9 |
| Tetanus | 11.5 |
| Idiopathic developmental intellectual disability | 11.4 |
| Conflict and terrorism | 11.0 |
| Exposure to forces of nature | 11.0 |
| Road injuries | 11.0 |
| Tracheal, bronchus, and lung cancer | 10.7 |
| Foreign body | 10.6 |
| Low back pain | 10.6 |
| Interpersonal violence | 10.3 |
| Ovarian cancer | 10.2 |
| Schizophrenia | 10.1 |
| Cervical cancer | 10.0 |
| Idiopathic epilepsy | 9.9 |
| Bipolar disorder | 9.7 |
| Falls | 9.4 |
| Fire, heat, and hot substances | 9.4 |
| Animal contact | 9.3 |
| Exposure to mechanical forces | 9.3 |
| Poisonings | 9.3 |
| Police conflict and executions | 9.3 |
| Kidney cancer | 9.2 |
| Prostate cancer | 9.0 |
| Pulmonary Arterial Hypertension | 9.0 |
| Uterine cancer | 9.0 |
| Decubitus ulcer | 8.7 |
| Other transport injuries | 8.7 |
| Environmental heat and cold exposure | 8.4 |
| Stomach cancer | 8.2 |
| Drowning | 8.1 |
| Oral disorders | 7.9 |
| Electrocution | 7.6 |
| Other unintentional injuries | 7.6 |
| Esophageal cancer | 7.1 |
| Pancreatic cancer | 6.7 |
| Chronic obstructive pulmonary disease | 6.4 |
| Larynx cancer | 6.2 |
| Lower extremity peripheral arterial disease | 5.8 |
| Gallbladder and biliary tract cancer | 5.7 |
| Bladder cancer | 5.6 |
| Motor neuron disease | 5.6 |
| Anxiety disorders | 5.4 |
| Headache disorders | 5.4 |
| Thyroid cancer | 5.4 |
| Lip and oral cavity cancer | 5.3 |
| Other pharynx cancer | 5.3 |
| Eating disorders | 5.1 |
| Osteoarthritis | 5.1 |
| Rheumatoid arthritis | 4.1 |
| Invasive Non-typhoidal Salmonella (iNTS) | 3.9 |
| Alcohol use disorders | 3.7 |
| Autism spectrum disorders | 3.7 |
| Cirrhosis | 3.6 |
| COVID-19 | 3.3 |
| Typhoid and paratyphoid | 3.3 |
| Cysticercosis | 3.0 |
| Leukemia | 2.9 |
| Malignant skin melanoma | 2.9 |
| Other malignant neoplasms | 2.9 |
| Scabies | 2.8 |
| Conduct disorder | 2.7 |
| Leprosy | 2.7 |
| Bacterial skin diseases | 2.5 |
| Hodgkin lymphoma | 2.1 |
| Iodine deficiency | 2.1 |
| Soft tissue and other extraosseous sarcomas | 2.1 |
| Neuroblastoma and other peripheral nervous cell tumors | 2.0 |
| Multiple myeloma | 1.9 |
| Testicular cancer | 1.9 |
| Malignant neoplasm of bone and articular cartilage | 1.8 |
| Gout | 1.7 |
| Eye cancer | 1.6 |
| Attention-deficit/hyperactivity disorder | 1.5 |
| Fungal skin diseases | 1.4 |
| Dermatitis | 0.9 |
| Nasopharynx cancer | 0.9 |
| Neck pain | 0.9 |
| Otitis media | 0.8 |
| Blindness and vision loss | 0.7 |
| Mesothelioma | 0.7 |
| Urticaria | 0.7 |
| Acne vulgaris | 0.4 |
| Age-related and other hearing loss | 0.4 |
| Vitamin A deficiency | 0.4 |
| Food-borne trematodiases | 0.2 |
| Dengue | 0.1 |
| Ebola | 0.1 |
| Other musculoskeletal disorders | 0.1 |
| Viral skin diseases | 0.1 |
| Zika virus | 0.1 |
| African trypanosomiasis | 0.0 |
| Alopecia areata | 0.0 |
| Aortic aneurysm | 0.0 |
| Dietary iron deficiency | 0.0 |
| Guinea worm disease | 0.0 |
| Hypertensive heart disease | 0.0 |
| Intestinal nematode infections | 0.0 |
| Lymphatic filariasis | 0.0 |
| Malaria | 0.0 |
| Onchocerciasis | 0.0 |
| Other cardiovascular and circulatory diseases | 0.0 |
| Other chronic respiratory diseases | 0.0 |
| Other digestive diseases | 0.0 |
| Other intestinal infectious diseases | 0.0 |
| Other mental disorders | 0.0 |
| Other neglected tropical diseases | 0.0 |
| Other neurological disorders | 0.0 |
| Other nutritional deficiencies | 0.0 |
| Other sense organ diseases | 0.0 |
| Other skin and subcutaneous diseases | 0.0 |
| Other unspecified infectious diseases | 0.0 |
| Pertussis | 0.0 |
| Protein-energy malnutrition | 0.0 |
| Pruritus | 0.0 |
| Psoriasis | 0.0 |
| Rabies | 0.0 |
| Sudden infant death syndrome | 0.0 |
| Trachoma | 0.0 |
| Upper respiratory infections | 0.0 |
| ^a^Coverage is defined as at least one available source providing relevant data; | |
